# Assessment of the use of different imaging and delivery techniques for cranial treatments on the Halcyon linac

**DOI:** 10.1002/acm2.12772

**Published:** 2019-11-18

**Authors:** Everardo Flores‐Martinez, Laura I. Cerviño, Todd Pawlicki, Gwe‐Ya Kim

**Affiliations:** ^1^ Department of Radiation Medicine and Applied Sciences University of California San Diego La Jolla CA USA

**Keywords:** AlignRT, Halcyon, rotational errors, SGRT, surface guided radiotherapy

## Abstract

**Purpose:**

In this work, we investigated the effect on the workflow and setup accuracy of using surface guided radiation therapy (SGRT) for patient setup, megavoltage cone beam CT (MVCBCT) or kilovoltage cone beam CT (kVCBCT) for imaging and fixed IMRT or volumetric‐modulated arc therapy (VMAT) for treatment delivery with the Halcyon linac.

**Methods:**

We performed a retrospective investigation of 272 treatment fractions, using three different workflows. The first and second workflows used MVCBCT and fixed IMRT for imaging and treatment delivery, and the second one also used SGRT for patient setup. The third workflow used SGRT for setup, kVCBCT for imaging and VMAT for delivery. Workflows were evaluated by comparing the number of fractions requiring repeated imaging acquisitions and the time required for setup, imaging and treatment delivery. Setup position accuracy was assessed by comparing the daily kV‐ or MV‐ CBCT with the planning CT and measuring the residual rotational errors for pitch, yaw and roll angles.

**Results:**

Without the use of SGRT, the imaging fields were delivered more than once on 11.1% of the fractions, while re‐imaging was necessary in 5.5% of the fractions using SGRT. The total treatment time, including setup, imaging, and delivery, for the three workflows was 531 ± 157 s, 503 ± 130 s and 457 ± 91 s, respectively. A statistically significant difference was observed when comparing the third workflow with the first two. The total residual rotational errors were 1.96 ± 1.29°, 1.28 ± 0.67° and 1.22 ± 0.76° and statistically significant differences were observed when comparing workflows with and without SGRT.

**Conclusions:**

The use of SGRT allowed for a reduction of re‐imaging during patient setup and improved patient position accuracy by reducing residual rotational errors. A reduction in treatment time using kVCBCT with SGRT was observed. The most efficient workflow was the one including kVCBCT and SGRT for setup and VMAT for delivery.

## 1 INTRODUCTION

In recent years there has been a growing interest in the assessment of the performance and quality of the treatment delivery process for external beam radiotherapy,^1^, ^2^, ^3^, ^4^ favoring a more efficient and safer clinical practice. In particular, time‐efficient workflows during radiotherapy treatments allow for a higher clinical throughput, improved patient comfort and reduced costs per treatments by reducing the machine and staff hours. Achieving such efficient workflows require a thorough assessment and optimization of each stage of the treatment process, including patient setup, image‐guided evaluation and delivery of the treatment fields.

The Halcyon^TM^ System (Varian Medical Systems, Palo Alto, CA) is a jawless, bore‐enclosed linear accelerator. Halcyon uses a 6 MV flattening filter free (FFF) beam for treatment and is streamlined for time‐efficient, daily, image‐guided radiation therapy (IGRT). For the version 1.0, orthogonal MV pairs or megavoltage cone beam CT (MVCBCT) imaging are available, while the version 2.0 also has kilovoltage cone beam CT (kVCBCT) capability. The dose contribution from the MV imaging fields is accounted for during plan optimization and dose calculation. The maximum gantry rotational speeds for the Halcyon gantry are 360° in 15 s, and 30 s for the IGRT, and treatment fields, respectively. Compared with the rotational speed of a C‐arm linac, Halcyon speeds are up to four and two times higher during the imaging and dose delivery stages, respectively. Such increased rotational gantry speeds, combined with a higher multileaf collimator (MLC) leaf speed allow for faster treatments. The tradeoff between plan quality and time efficiency in Halcyon has been investigated for some sites as prostate^5^ and head and neck.^6^ In both cases, the plan quality with Halcyon was maintained but the imaging and dose delivery times were reduced as compared with treatments using a C‐arm linac.

In a typical treatment with Halcyon, the patient is aligned to the virtual isocenter, a reference point outside the bore, and then, the couch is moved into the bore to the treatment isocenter. Image registration to the CT is performed using a 2D‐3D image registration when the orthogonal pairs are used, or a 3D‐3D registration when the CBCT technique is used, to calculate and apply the shifts between the CT and daily images. The couch in Halcyon only allows for translational shifts, and, if large rotations are observed on the initial images, the patient needs to be manually re‐aligned and additional images are taken. Several authors have reported the dosimetric consequences of rotational setup errors.^7^, ^8^, ^9^, ^10^ Peng et al. reported changes up to 11% on the clincal target volume (CTV) coverage for cranial stereotactic radiosurgery (SRS),^7^ and Briscoe showed that setup accuracy is especially critical for plans treating multiple metastases with a single isocenter.^10^ Initial setup accuracy is relevant, not only to ensure accurate dose delivery, but also to improve the workflow and prevent excess imaging fields. The time for correcting the setup and taking new images is added to the total treatment time, thus decreasing the efficiency of the process and worsening the patient experience as he or she has to spend more time on the couch.

Surface Guided Radiation Therapy (SGRT) provides an option for improved patient setup and real‐time monitoring and has been used previously for intracranial^11^, ^12^, ^13^, ^14^ and extracranial regions.^15^, ^16^, ^17^, ^18^, ^19^, ^20^, ^21^ SGRT is a nonradiographic localization technique that uses a system of 3D cameras to detect a light pattern projected onto the patient and use the information to reconstruct a 3D surface image. The generated 3D image can be compared real time to the body contour from the CT scan at simulation and assist with patient setup and tracking.

The purpose of this work was to evaluate the optimal imaging modalities and dose delivery techniques, for cranial treatments using the Halcyon linac considering workflow efficiency and setup accuracy.

## 2 METHODS AND MATERIALS

At our institution, non‐SRS cranial treatments are performed using the Halcyon linac. For this set of patients, all undergoing standard fractionations and with doses per fraction ranging from 1.8 Gy to 3.0 Gy, the planning simulation is performed with a General Electric computed tomography G scanner using a slice thickness of 2.5 mm and immobilization is achieved by using a head rest and a full mask. In this work, we compared retrospectively the efficiency and setup accuracy of three workflows using different setup, imaging and dose delivery modalities.

The first and second workflows correspond to patients treated with version 1.0 of Halcyon and used MVCBCT for imaging and fixed IMRT for delivery. Additionally, the second workflow also used SGRT for patient setup. The third workflow corresponds to patients treated with the version 2.0 of Halcyon and used SGRT for setup, kVCBCT for imaging and volumetric‐modulated arc therapy (VMAT) for delivery.

**Figure 1:**
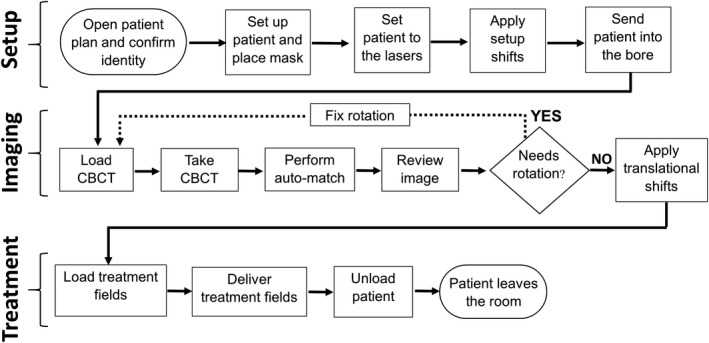
Pre‐SGRT workflow for cranial treatments in the Halcyon linac showing the three main stages during a session: Setup, imaging and treatment. SGRT, surface guided radiation therapy.

The workflow to treat patients with intracranial lesions using Halcyon consists of three main stages: setup, imaging, and treatment. The first stage starts when the patient enters into the room, is set up on the couch to the virtual lasers, immobilized by using the full mask, and finally sent into the bore to the treatment isocenter. The second stage includes the steps necessary to verify and adjust the patient setup by using image‐guidance. The IGRT fields are delivered and the corresponding images are checked, and, if large rotations are observed, the therapist goes back into the room and adjusts the patient. After adjustment, new IGRT fields are taken and the necessary translational shifts are applied. The third stage in the workflow includes the delivery of the treatment fields and finishes when the patient leaves the room. Figure 1 shows the workflow at our institution for intracranial treatments before the implementation of SGRT.

### 2.1 SGRT implementation

**Figure 2:**
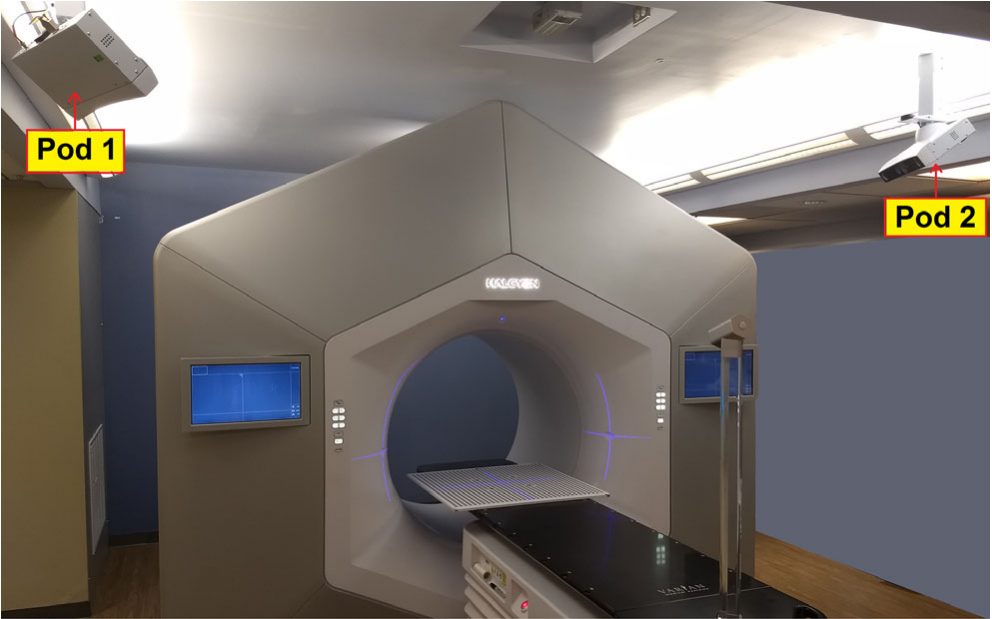
Configuration of the SGRT pods on the Halcyon vault and the circle pattern used for daily and monthly calibration. SGRT, surface guided radiation therapy.

**Figure 3:**
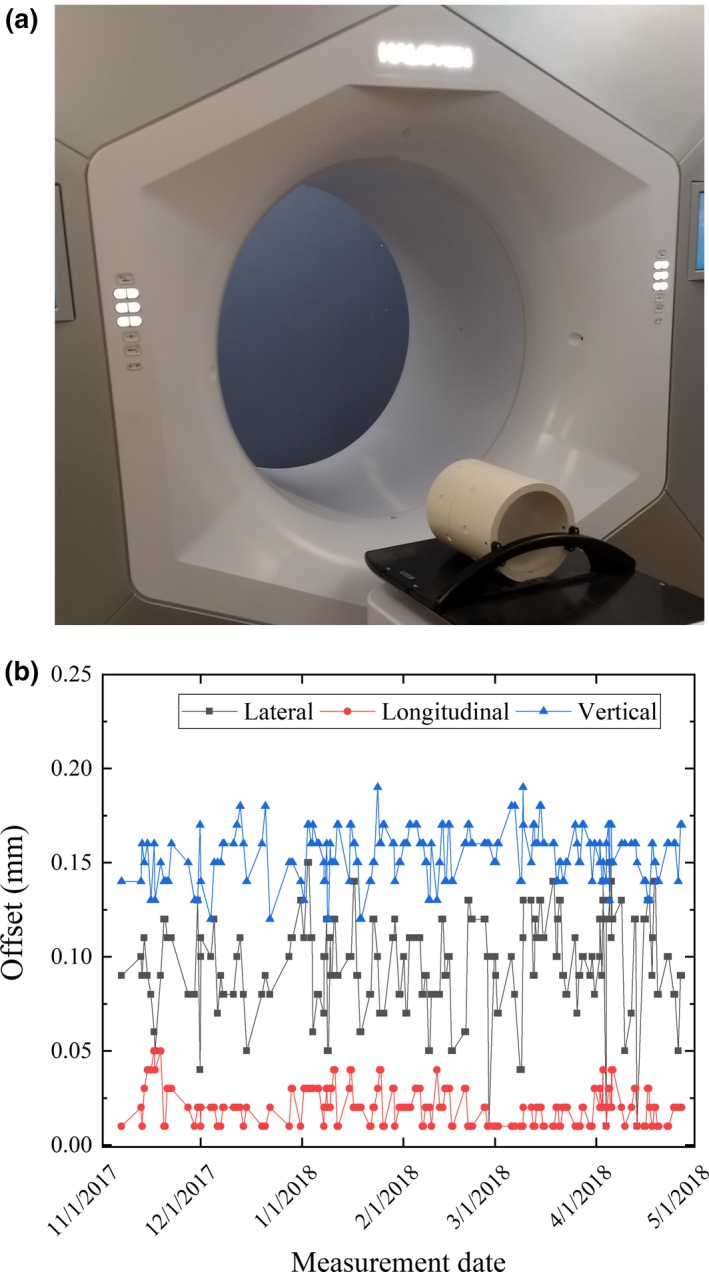
(a) Drum phantom used for daily MPC on Halcyon. (b) Measured offset between virtual and treatment isocenter as reported by the daily machine performance check (MPC) during the span of this work.

**Figure 4:**
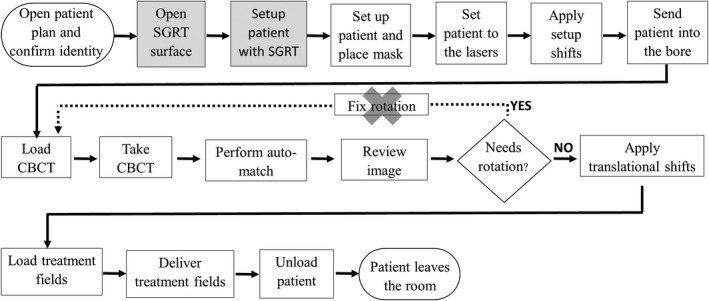
Workflow for cranial treatments in Halcyon using SGRT. SGRT‐related steps have been highlighted. SGRT, surface guided radiation therapy.

AligntRT^®^ (Vision RT Ltd, London UK) is a video‐based 3D camera system used for SGRT. Figure 2 shows the configuration for AligntRT at the Halcyon vault on our clinic, consisting of a two‐camera system, located at the ceiling and aligned to the virtual isocenter defined by the lasers out of the bore. Daily calibration is performed by using a plate with a circle pattern. For each camera, the system detects reference markers, whose locations are refined by the user, and determines the isocenter. Unlike conventional implementations of AlignRT, the two‐camera system on the Halcyon linac cannot be calibrated to the treatment isocenter due to blockage by the gantry bore. Therefore, the calibration is performed using the virtual isocenter. The coincidence between the virtual and treatment isocenter is measured by an integrated self‐check tool, the machine performance check (MPC). MPC is enforced every day before treatment and requires a drum phantom on the couch [Fig. 3(a)]. The drum phantom is set at a fixed position every day, and the user verifies the laser’s accuracy by checking coincidence with marks in the phantom. The offset between the virtual isocenter and the machine isocenter is measured during MPC, acquiring images, identifying landmarks in the phantom and measuring the shifts in the registration. Figure 3b shows longitudinal offset measurements at our institution during the span of this work. In order to incorporate the use of SGRT, the workflow was modified adding one step for loading the patient and another to setup the patient using SGRT (Fig. 4). Currently, there is no surface tracking after the thermoplastic mask is placed.

### 2.2 Phantom‐based accuracy verification

The accuracy of the method used in this work to measure the residual rotational errors was evaluated using the AlignRT isocenter calibration phantom, which has five embedded ceramic spheres and is mounted on a base with three leveling screws. The phantom was scanned with a GE CT scanner using a head protocol with 1.25 mm slices and a surface image was generated using the Eclipse™ treatment planning system (Varian Medical Systems, Palo Alto CA). The phantom was set to the virtual isocenter in Halcyon and rotational offsets from −1.5° to 1.5° on 0.5° steps were induced for pitch, yaw and roll using the leveling screws on the base. The rotational shifts were verified using AlignRT and a digital level, ensuring only one non‐zero angular rotation per measurement. With the phantom at the machine isocenter, a CBCT scan was acquired and imported into MIM version 6.7.4 (MIM software Inc, Cleveland OH). Residual rotational errors were calculated by performing an automatic rigid registration between the CBCTs and the original CT using MIM. The difference between induced and measured angles was determined and used to estimate the accuracy of the technique.

### 2.3 Data acquisition

Approval for the study was granted by the University of California San Diego Institutional Review Boards, project #181861XL. Table 1 shows the demographics for the fractions investigated in this work. Plans investigated included treatment sites such as orbits, head and neck, glioblastoma multiple (GBM) and brain, with treatment volumes ranging from 9.5 cm^3^ to 456 cm^3^. Data for 272 fractions, distributed among 15 patients, were analyzed. Five metrics were used to evaluate the impact over the workflow and dose delivery accuracy of different setup, imaging and delivery modalities:

- Percent of fractions with additional imaging. The number of images that were acquired in addition to the planned images because the patient had to be manually repositioned after the initial setup. We compared 117 fractions without SGRT with 155 fractions using SGRT.
- Setup time. Starting at the instant when the patient was loaded into the system and ending when the first imaging field was delivered, it was measured by using the timestamps in the record and verify system (Aria^®^ V15.5, Varian Medical Systems, Palo Alto, CA).
- Imaging time. Including the delivery of the first imaging field, the evaluation of the image registration and, the application of the necessary couch shifts and ending at the delivery of the first treatment field, measured using the timestamps on the record and verify system. For the fractions treated with Halcyon 1.0 (117 without SGRT and 107 with SGRT), the imaging technique was MVCBCT. Fractions delivered using version 2.0 (48 fractions) used kVCBCT with iterative reconstruction as the imaging technique.
- Treatment time. The overall time for therapeutic dose delivery. It was measured retrospectively using the timestamps provided by the record and verify system. The field delivery technique with Halcyon 1.0 was fixed IMRT, usually using nine fields and for Halcyon 2.0, the delivery technique changed to VMAT.
- Magnitude of residual rotational errors. Measured by comparing the rotation between the daily images and the CT scan from simulation. Each of the daily CBCTs was exported into the MIM workspace, fused with the simulation CT scan and the residual rotational errors for yaw, pitch and roll were obtained from the automatic registration results. A rigid box‐based alignment was used with a region of interest including the base of skull and a bone windowing level. The composite angle deviation was calculated as the square root of the sum of the squares for the three rotation angles.

**Table 1:** Types of plan and number of fractions for treatment and delivery techniques investigated.

Site	PTV volume (cm^3^)	MVCBCT, Fixed IMRT & without SGRT	MVCBCT, Fixed IMRT & with SGRT	kVCBCT, VMAT & with SGRT
Brain				
B1	243.6	15	0	0
B2	305.1	32	0	0
B3	426.0	0	0	11
B4	195.9	0	0	14
B5	132.1	0	0	8
B6	27.1	0	0	5
GBM				
G1	221.9	10	10	0
G2	207.4	0	30	0
G3	255.7	4	11	0
G4	239.4	0	30	0
G5	295.2	0	0	10
H&N				
H1	456.0	29	4	0
Orbit				
O1	47.9	7	10	0
O2	9.5	0	12	0
O3	25.9	20	0	0
Total		117	107	48

All data were analyzed calculating the mean, median, maximum and quartile distributions. The nonparametric Mann‐Whitney test was used to establish the statistical significance of the results at *P* < 0.01 level.

## 3 RESULTS

### 3.1 Phantom‐based accuracy verification

**Figure 5:**
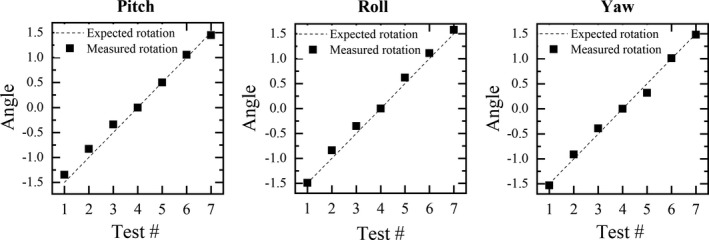
Residual errors measured after inducing known rotations on the AlignRT isocenter phantom.

Figure 5 shows the results for the phantom‐based evaluation. The maximum difference between induced and the measured rotational shifts was less than 0.2° and the mean squared errors for pitch, jaw and roll were 0.11°, 0.09° and 0.11°, respectively.

### 3.2 Time for setup, imaging and treatment fields

**Figure 6:**
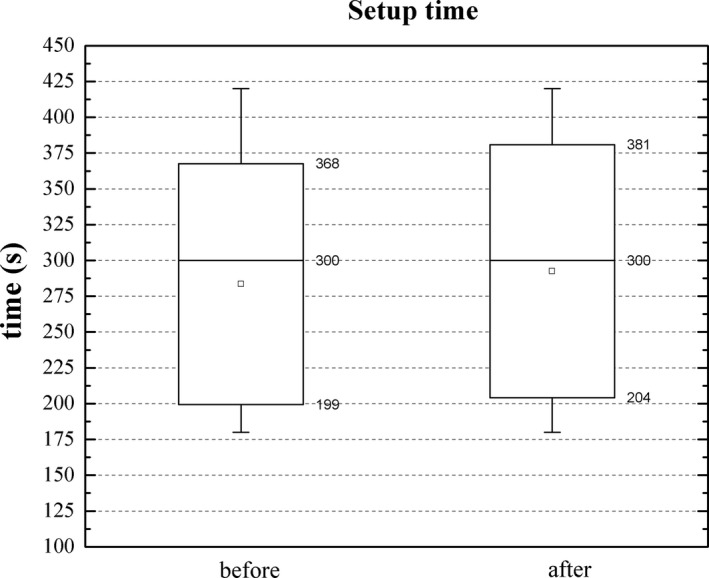
Setup time before and after the use of SGRT. The mean (squares) and median (horizontal line) values are shown. The top and bottom of the boxes indicate the 25% and 75% quartiles and the whiskers show the 10–90% interval. SGRT, surface guided radiation therapy.

Without the use of SGRT, additional imaging fields were necessary in 11.1% of the fractions. Once SGRT was incorporated into the workflow, the percentage of fractions requiring more than one imaging field was reduced to 5.5% (Table 2). Figure 6 shows the results for the setup time with and without SGRT. Setup time for patients treated before the implementation of SGRT was 283 ± 84 s, and with the use of SGRT, the mean time was 293 ± 89 s.

**Table 2:** Comparison of results before and after implementing SGRT. Stated values indicate mean ± SD and statistical significance is shown in parenthesis, p‐values were calculated by comparing with data in the first column.

Metric	MVCBCT, fixed IMRT & without SGRT	MVCBCT, fixed IMRT & with SGRT	kVCBCT, VMAT & with SGRT
Percent of fractions with multiple imaging fields	11.1%	5.5%	
Setup time (s)	283 ± 84	293 ± 89 (NS)	
Imaging time (s)	112 ± 95	89 ± 46 (NS)	97 ± 43 (NS)
Delivery time (s)	143 ± 26		119 ± 30 (*P* < 0.01)
Pitch (°)	1.24 ± 0.97	0.53 ± 0.45 (*P* < 0.01)	0.60 ± 0.38 (*P* < 0.01)
Yaw (°)	0.93 ± 0.98	0.79 ± 0.66 (NS)	0.60 ± 0.59 (NS)
Roll (°)	0.80 ± 0.76	0.54 ± 0.49 (NS)	0.63 ± 0.71 (NS)
Total angle (°)	1.96 ± 1.29	1.28 ± 0.67 (*P* < 0.01)	1.22 ± 0.76 (*P* < 0.01)

SGRT, surface guided radiation therapy.

**Figure 7:**
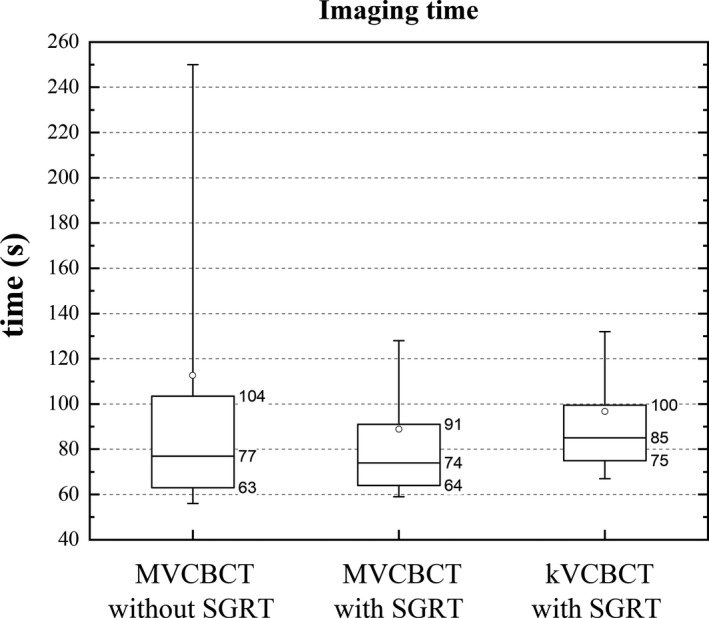
Imaging time with and without the use of SGRT using MVCBCT fields, also shown, data with SGRT and kVCBCT. The mean (squares) and median (horizontal line) values are shown. The top and bottom of the boxes indicate the 25% and 75% quartiles and the whiskers show the 10‐90% interval. SGRT, surface guided radiation therapy.

The average time for the imaging stage, using MVCBCT fields, was 113 ± 95 s and 89 ± 46 s before and after the implementation of SGRT, respectively (Fig. 7). For the fractions delivered using the kVCBCT technique and SGRT, the mean imaging time was 97 ± 43 s.

**Figure 8:**
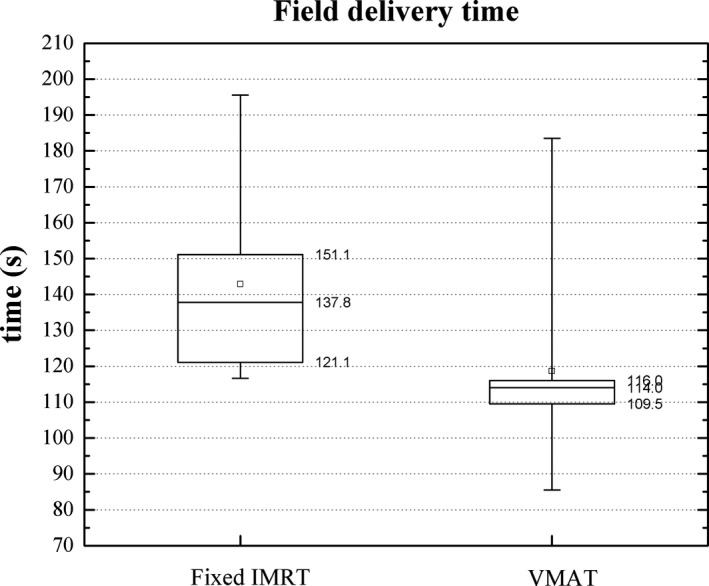
Field delivery time for intracranial treatments using fixed IMRT and VMAT. The mean (squares) and median (horizontal line) values are shown. The top and bottom of the boxes indicate the 25% and 75% quartiles and the whiskers show the 10–90% interval.

For the delivery of the treatment fields, there was no difference in the workflow with or without the use of SGRT and thus, no variation was anticipated at this stage. After the update to version 2.0, treatments were delivered using VMAT instead of fixed IMRT changing the field delivery time. Figure 8 shows the comparison in the delivery time between these two delivery techniques. Data on the upper quartiles correspond either to more modulated plans or, in the case of VMAT, treatments with four arcs. The mean delivery time was 143 ± 26 s and 119 ± 30 using fixed IMRT and VMAT, respectively.

**Figure 9:**
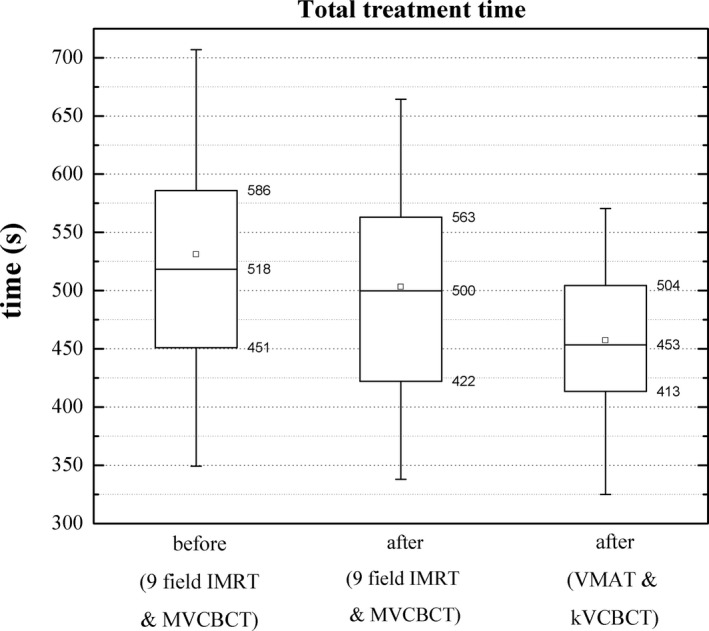
Total treatment time before and after the use of SGRT using MVCBCTs and fixed IMRT. Also shown, total treatment time using SGRT for treatments with kVCBCT and VMAT. The mean (squares) and median (horizontal line) values are shown. The top and bottom of the boxes indicate the 25% and 75% quartiles and the whiskers show the 10–90% interval. SGRT, surface guided radiation therapy.

Figure 9 shows the overall times when all the stages are considered. The average total treatment time using version 1.0 of Halcyon (MVCBCT and fixed IMRT) was 531 ± 157 s and 503 ± 130 s with and without SGRT, a difference that was not statistically significant (*P* = 0.227). For fractions treated using Halcyon version 2.0 and SGRT, the mean value was 457 ± 91 s, a statistically significant difference when compared with treatments using version 1.0 and without SGRT (*P* = 0.002).

**Figure 10:**
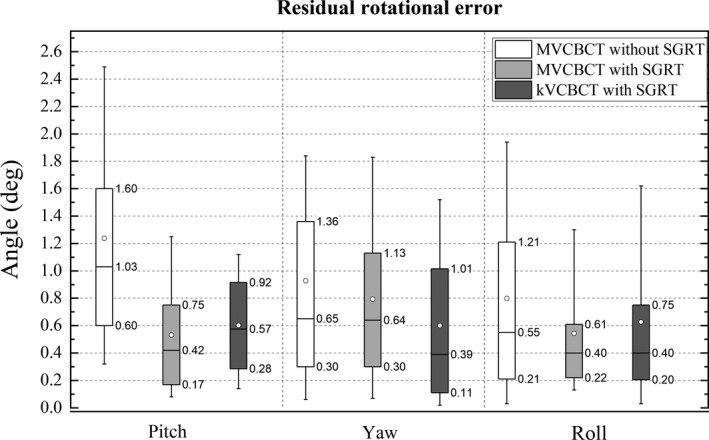
Residual rotational errors with and without SGRT. The mean (white squares) and median (horizontal line) values are shown. The top and bottom of the boxes indicate the 25% and 75% quartiles and the whiskers show the 10–90% interval. SGRT, surface guided radiation therapy.

Figure 10 shows the residual rotational errors with and without SGRT, as compared by the registration of the daily CBCT fields with the CT at simulation. The mean absolute residual rotational errors for pitch, yaw and roll without SGRT were 1.24 ± 0.97°, 0.93 ± 0.98° and 0.80 ± 0.76°, respectively, for fractions using MVCBCT and without SGRT. For treatments using MVCBCT and SGRT, the residual rotational errors were 0.53 ± 0.45°, 0.79 ± 0.66° and 0.54 ± 0.49°. The Mann‐Whitney test showed a statistical significant difference for the pitch (*P* < 0.001) but not for the yaw (*P* = 0.632) or the roll (*P* = 0.045). For treatments using kVCBCT and SGRT the residual rotational errors for pitch, yaw and roll were 0.60 ± 0.38°, 0.60 ± 0.59° and 0.63 ± 0.71°, respectively. Compared with the fractions using MVCBT and without SGRT the residual rotational errors for pitch were statistically significant different (*P* < 0.001) but not the yaw (*P* = 0.023) or roll (*P* = 0.173).

## 4 Discussion

Phantom measurements allowed to establish a reference to evaluate the accuracy of the technique to measure rotational shifts. The difference between induced and measured rotational shifts was less than ± 0.2°. A limitation of this characterization is that phantom and patient geometry are different in terms of high contrast regions used for the auto‐registration. For the phantom, the registration is mainly driven by matching the five ceramic spheres embedded in the phantom, while for the patient images, the bony structure from the skull is used. A similar study, using a home‐made cranial phantom, performed by Mancosu et al.^22^ reported an estimated maximum rotational inaccuracy of ± 0.3° for SGRT‐based setups.

SGRT implementation allowed for a reduction up to 50% in the number of fractions requiring setup correction and additional imaging fields. This reduction is particularly relevant for a patient‐centered machine like Halcyon, streamlined for high‐throughput and fast patient treatment and it also reduces schedule delays on the machine and excessive iterations on patient setup.

The use of SGRT increased the mean setup time by about 10 s. The factors that contribute to increase this time are the additional time to turn on the AlignRT cameras and variations in time the therapists require to align the patient either with the lasers or using SGRT. This difference, however, was found not to be statistically significant according with the Mann‐Whitney test (*P* = 0.486). While the Mann‐Whitney tests showed no statistical difference for the imaging time using MVCBCT (*P* = 0.421), the standard deviation, an indicator of the inter‐fraction and inter‐patient variability was reduced from 95 s to 46 s after the SGRT implementation. The time for data on the first two quartiles was similar with and without SGRT but significant differences were observed for data on the 90th percentile (Fig. 7). When comparing MVCBCT and kVCBCT fractions, both using SGRT, differences can arise from the speed of the reconstruction, automatic registration and image quality that allows a faster review and correction of the registration by the therapist. While the mean imaging time for kVCBCT cases was 8 s higher than the time using MVCBCT, this difference was not statistically significant (*P* = 0.083). More relevant factors to choose kVCBCT over MVCBCT are less imaging dose and better image quality.

Regarding the time for the delivery of the treatment fields, the difference between fixed IMRT and VMAT was found to be statistically different (*P* = 0.0042), a result consistent with previous works comparing both techniques for other treatment sites.^23^, ^24^ Lessening the radiotherapy treatment time allows for higher patient throughput and also improves patient comfort by reducing the time lying on the treatment table.

The use of SGRT provides an additional layer of safety for patient setup and allows for a significant reduction on the total residual rotational errors at the *P* < 0.01 level. In this way, SGRT ensures a more accurate delivery of the radiation dose at every fraction. The quantification of the dosimetric impact of this improvement requires further investigation and is expected to be more relevant when the treated area is closer to organs at risk.

In this work we have shown the advantages of using SGRT for initial setup of cranial treatments using the Halcyon linac. However there are other sites that could also benefit from this technique such as head and neck, extremities, breast and prostate as has been shown for C‐arm linacs.^15^, ^16^, ^17^, ^18^, ^19^, ^20^, ^21^ We identified two limitations of the current SGRT setup. First, the use of full masks precludes surface tracking after mask placement. It is possible that some of the patients move during mask placement and such deviations are not detected prior to the setup fields. A second limitation is that the cameras are calibrated to the virtual isocenter and do not monitor the patient during the treatment. Future developments on SGRT capable to monitor the patient at the radiation isocenter will allow monitoring intrafractional motion by real‐time tracking.

## 5 CONCLUSIONS

We have shown that the most efficient workflow for patients treated in the cranial region using Halcyon is the one that uses SGRT for patient setup, kVCBCT for imaging and VMAT for delivery. The use of SGRT allows for a reduction over the number of additional imaging fields on the patient setup, and a more accurate dose delivery by means of a significant reduction of the residual rotational error.

## CONFLICT OF INTEREST

UC San Diego received in‐kind funding from Vision RT Ltd in partial support of this research project.
